# Electrophysiological correlates of contextual retrieval following virtual reality and desktop-based encoding

**DOI:** 10.3389/fnhum.2026.1866317

**Published:** 2026-10-14

**Authors:** Joanna Kisker, Merle Sagehorn, Thomas Gruber

**Affiliations:** Experimental Psychology I, Institute of Psychology, Osnabrück University, Osnabrück, Germany

**Keywords:** contextual retrieval, ERP old/new effects, recognition memory, source memory, virtual reality

## Abstract

Virtual reality (VR)-based encoding has been proposed to improve episodic memory retrieval compared to desktop (PC)-based encoding, but behavioral evidence remains inconsistent. Potentially, VR- and PC-based encoding affect the retrieval processes involved in recognition differently, rather than overall memory performance. Building on prior research, the present study investigated whether memory retrieval incorporates the encoding modality (VR vs. PC) as contextual information bound to the stimulus when accounting for additional task-related contextual information. To this end, participants encoded everyday objects in both VR and PC settings, with the encoding task varying across objects. A later recognition memory test involved old/new judgments of the encoded objects and source identifications of the encoding modality and task. Memory performance was similar across encoding modalities and relatively low, showing no advantage for recognition or source attribution following VR-based encoding. At the neural level, both VR- and PC-based memory traces elicited similar FN400 old/new effects, indicating comparable familiarity-related contributions. The LPC old/new effect was significant only for VR-encoded items, whereas the direct VR–PC comparison was not. Source-sorted LPN analyses yielded slightly different patterns descriptively, but the direct comparison did not establish a difference between modality- and task-source sorting. Although direct modality-specific effects were limited, the results are compatible with previous studies indicating that VR-based encoding may be associated with relatively greater recollection-related retrieval processes.

## 1 Introduction

Memories of everyday experiences are mainly stored in and retrieved from episodic memory, a fundamental system that shapes future behavior, decisions, and experiences (Conway, 2005; Tulving, 1983, 1985). Immersive technologies such as Virtual Reality (VR) provide new ways to experience and encode information, raising the question of how we acquire, process, and remember that information. However, the impact of immersive technologies such as VR on episodic memory remains a matter of debate, especially compared with conventional two-dimensional screens (PCs). It has been proposed that episodic memory for VR experiences is improved relative to PC experiences (for a review, see e.g., Smith, 2019; Smith and Mulligan, 2021). This hypothesis stems from VR’s immersive characteristics, which integrate spatial and temporal features into a sensory-rich environment, regarded as fundamental to episodic memory (Parsons, 2015; Serino and Repetto, 2018; Smith, 2019; Smith and Mulligan, 2021). These characteristics raise the question of whether information encoded in VR is remembered differently from information encoded on a PC. Most previous studies have addressed this question on a behavioral level by comparing retrieval success after VR- and PC-based encoding. On the one hand, encoding in VR tends to result in higher retrieval rates than encoding the same material in PC-based settings (memory superiority effect; Harman et al., 2017; Schöne et al., 2019, 2021; for reviews see Smith, 2019; Smith and Mulligan, 2021). However, this memory superiority is not reliably replicated. Some findings indicate similar memory performance in both conditions (Cadet and Chainay, 2020; Ernstsen et al., 2019; Johnsdorf et al., 2025; Kisker et al., 2021b; Kisker et al., 2021a; Kisker et al., 2025c) or even superior retrieval after encoding on a PC (Johnsdorf et al., 2023; Makransky and Petersen, 2021). Thus, the putative superiority of VR-based memory traces in retrieval remains a matter of debate.

Successful recognition observed in behavioral outcomes can generally depend on familiarity, recollection, or their combination. Both processes contribute in complementary ways to recognition memory as proposed in the dual-process theory of recognition memory (Rugg and Curran, 2007; Tulving, 1985; Yonelinas, 2002). Familiarity refers to an automatic retrieval process that provides a general sense of recognition (Rugg and Curran, 2007; Yonelinas, 2002). In contrast, recollection is defined as conscious retrieval of contextual details from the encoding episode, such as where or how an item was encountered. It is considered a more controlled process compared with familiarity (Diana et al., 2008; Eichenbaum et al., 2007; see also Rugg and Curran, 2007; Yonelinas, 2002).

Although familiarity and recollection complement each other, their roles in retrieval can vary, and successful recall may result from different combinations of these processes. One possible explanation for the inconsistent behavioral results is that encoding in VR versus on a PC differently affects the balance between familiarity and recollection (Johnsdorf et al., 2025; Kisker et al., 2021b; Kisker et al., 2021a; Kisker et al., 2025c; Schöne et al., 2019). In particular, accumulating evidence links retrieval following VR-based encoding to relatively greater contributions of recollection than of familiarity, whereas retrieval following PC-based encoding is thought to be linked to greater contributions of familiarity than of recollection (Kisker et al., 2021b; Kisker et al., 2021a; Kisker et al., 2025c). Whereas early studies differentiated both processes based on subjective awareness of and access to these processes (Kisker et al., 2021b; Kisker et al., 2021a), most recent findings objectively discriminated between both processes by explicitly testing for contextual information from the encoding episode retrieved with the item, i.e., source memory (Kisker et al., 2026; Kisker et al., 2025c). Source memory refers to remembering the context in which an item was encoded, like spatiotemporal details (Bröder and Meiser, 2007) or the sensory encoding modality (Johnson et al., 1993). Recalling a stimulus along with its source is regarded as a hallmark of recollection, whereas recognizing a stimulus without recalling its source indicates greater reliance on familiarity (Migo et al., 2012). These associations enable source memory paradigms to objectively differentiate between familiarity and recollection (Koen and Yonelinas, 2014; Yonelinas and Jacoby, 2012).

These assumptions raise the question of whether the encoding modality itself, here VR versus PC, becomes part of the remembered context. In that case, the encoding modality might be a key factor in recollection-based recognition performance. Preliminary findings from VR, PC, and real-life settings indicate that the encoding modality is processed as part of the encoding context and can, in principle, be accessed during retrieval (Bonnail et al., 2024; Rubo et al., 2021). While these initial findings are based on behavioral retrieval results rather than dissociating the underlying processes, a more recent study (Kisker et al., 2025c) examined whether neural activity patterns support the assumption that the encoding modality can later be remembered as source information. In that case, the encoding modality might facilitate differences in retrieval processes between VR-based and PC-based memory traces (hereafter: VR-traces; PC-traces). This effect might potentially be independent of the specific information encoded within either modality (Kisker et al., 2025c). In this study, participants encoded everyday objects in VR or on a PC, followed by a recognition memory task that included old/new judgments to examine item memory and source identification (VR/PC) to assess source memory. Familiarity and recollection were examined by analyzing specific deflections of the event-related potential (ERP) in the electroencephalogram (EEG). Familiarity-related recognition is often linked to a frontal old/new effect around 300–500 ms, commonly referred to as the FN400. The FN400 is usually less negative-going in response to old stimuli than to new stimuli (Kwon et al., 2023; Mecklinger, 2000; Rugg and Curran, 2007). Conversely, recollection-related processing is associated with a parietal old/new effect around 500–800 ms, the late positive component (LPC; Johansson and Mecklinger, 2003; Kwon et al., 2023; Rugg and Curran, 2007). The LPC usually occurs when the retrieval cue elicits a relatively spontaneous, stimulus-driven recollection response (Kamp et al., 2025). Complementarily, the search for source-specific attributes is associated with the late posterior negativity (LPN; Cycowicz et al., 2001; Herron, 2007; Johansson and Mecklinger, 2003; Kamp et al., 2025; Mecklinger et al., 2016). Different accounts of the LPN’s functional significance vary in their focus on the search, the reconstruction, or the amount of contextual detail available from the encoding context during retrieval. However, they overlap in referring to the retrieval of contextual information associated with the encoding episode (see, e.g., Addante et al., 2024; Johansson and Mecklinger, 2003; Mecklinger et al., 2007, 2016). In contrast to the LPC, the LPN is associated with more effortful and controlled processes that mediate the deliberate reconstruction of contextual details of the memory trace’s source (Kamp et al., 2025).

The preceding study’s (Kisker et al., 2025c) results indicated that encoding modality has a limited impact on item memory. Specifically, no significant differences between VR- and PC-traces in recognition performance or in the FN400 and the LPC were observed. In contrast, the LPN results suggested that source-related retrieval may differ between VR- and PC-encoded items. As a relative index linked to recollection-related activity, the LPN amplitudes for items with correct source attributions were compared with those for items with incorrect source attributions. Similarly, items with incorrect source attribution were compared with correctly rejected new items as a relative index linked to familiarity-related activity. This approach aimed to account for the proportion of the response related to correct recognition in the absence of accurate contextual retrieval (see also Kwon et al., 2023). These contrasts indicated relatively greater contributions of recollection and relatively weaker contributions of familiarity to the retrieval of VR-traces, particularly when source retrieval failed. These results suggested that the encoding modality could generally be retrieved in conjunction with the item. Moreover, they indicated that familiarity-related activity may be sensitive to perceptual or contextual differences between VR- and PC-based encoding (Kisker et al., 2025c).

The current study addresses two limitations of the preceding study. First (I), it examines both early and later LPN time windows. Second (II), it assesses source information beyond the encoding modality. (I) The LPN is linked to slightly different processes depending on its specific timing. The early phase of the LPN deflection (~ 700–1,200 ms after stimulus onset) is more closely related to the immediate retrieval process; its later stages are associated with source monitoring and post-retrieval processing (~1,200–1800 ms after stimulus onset; Herron, 2007; Johansson and Mecklinger, 2003; Mecklinger et al., 2007, 2016). In the previous study, the LPN was examined only in the earlier time window associated with immediate retrieval. Its later stages, associated with source monitoring and post-retrieval processing, were not examined because the response was confounded with stimulus-offset-related activity. Consequently, the current study design adapted the stimulus presentation to examine whether analyses of the later LPN stage yield similar differences between VR- and PC-traces as in the earlier stage. (II) Even more importantly, the preceding study specifically explored whether the encoding modality served as relevant contextual information and, for this purpose, tied source memory exclusively to the successful retrieval of that modality, with no further contextual information tested. Thus, the previous task may have missed other remembered details that were not directly tested, often referred to as non-criterial recollections (Johnson et al., 1993; Yonelinas and Jacoby, 1996). However, participants might generally recall the encoding modality (VR/PC), additional information beyond the encoding modality, or both. As a result, source accuracy may depend on which type of source information is tested. Given the information the task targets, the procedure might lead to an underestimation of recollection or an overestimation of familiarity, respectively (Parks, 2007; Yonelinas and Jacoby, 1996). Assessing different source types might therefore clarify whether memory depends mainly on the encoding medium itself or rather on other aspects of the encoding episode.

Since the encoding modality was not directly relevant for the encoding task in the preceding study, non-critical recollection of information more closely tied to the encoding task might have been overlooked. This study consequently extends prior insights by assessing source information beyond the encoding modality. According to the levels of processing model (Craik and Lockhart, 1972), information about sensory or physical features is processed relatively early or shallowly. In contrast, processing the semantic meaning of stimuli affords higher cognitive functions and thus leads to deeper processing (Craik and Lockhart, 1972). While differences in the encoding modality might predominantly affect initial processing steps, they might also interact with further contextual information associated with the encoding episode. For example, estimations of an object’s size might be facilitated by stereoscopic information. Traditional source memory paradigms vary in whether they afford retrieval of perceptual details visible during encoding, such as stimulus location (Glisky et al., 2001), or semantic aspects of the item, such as its real-world size or whether it is animate (Gruber et al., 2008). Building on these classic tasks, participants in the current study completed one of two encoding tasks for each stimulus—either categorizing its real-life size or its position within the virtual environment. In the subsequent source identification task, both the encoding modality and the encoding task were assessed. Although this approach cannot capture all remembered contextual information, it enables initial insights into different types of source information assessed at retrieval of the same encoding episode.

Based on this rationale, the current study examined whether previously observed modality-related LPN effects (Kisker et al., 2025c) were more closely associated with later modality-source accuracy or whether they may also reflect other remembered details from the encoding episode. Alternatively, they may have been affected by non-criterial recollections, which were not considered in the previous study. To this end, we included two source judgments after the old/new decision: one assessing the encoding modality and one assessing the encoding task. If previously reported LPN effects (Kisker et al., 2025c) were affected by non-criterial recollection, similar LPN patterns should emerge when the same retrieval-cue-locked ERPs are sorted according to later modality-source and task-source accuracy. Conversely, if the encoding medium is a distinct source detail, another overall pattern of results may be associated with later modality-source accuracy than with later task-source accuracy. At the same time, we expect similar FN400 and LPC old/new effects for VR- and PC-traces, indicating that item retrieval per se depends on both well-established processes of the dual-process accounts. Since the preceding study did not reveal differences in memory performance across encoding modalities despite differences in memory processes, we do not expect differences in behavioral estimates of memory performance as well.

## 2 Methods

### 2.1 Sample size and participants

The study procedures were approved by the ethics committee of Osnabrück University (Ethik-36/2024). Participants gave informed written consent and were unaware of the research question and hypotheses. They were compensated with partial course credits or monetary compensation. G* Power (Faul et al., 2007) was used for a power analysis. The analysis focused on a 2 × 5 repeated-measures ANOVA (rmANOVA) with an estimated effect size of *f =* 0.37, derived from an rmANOVA on the LPN with *η_p_^2^* = 0.12 (Kisker et al., 2025c). It indicated a minimum sample size of 10 participants. Because this estimate was derived from a relatively large LPN effect observed in prior work and did not fully account for component-specific data-quality exclusions in the present study, it should be considered an optimistic estimate for smaller modality-specific effects. We therefore targeted a substantially larger sample of approximately 30 participants, which is consistent with sample sizes commonly used in within-subject ERP studies (Clayson et al., 2019) and exceeds the estimated minimum sample size by a considerable margin. Ultimately, 34 participants were recruited from Osnabrück University to account for potential technical issues and align with previous VR research practices. All participants were screened for psychological or neurological issues. Two participants withdrew before data collection, and one was excluded for not meeting inclusion criteria, leaving 31 participants (*M*_age_ = 21.90; *SD*_age_ = 2.77; 87.1 right-handed, 3.2% no dominant hand; sex: 80.6% female, 19.4% male; all cisgender). Among these, 80.6% had prior VR experience with head-mounted displays (HMD) but were not regular users. 48.4% used computers regularly for gaming or entertainment.

### 2.2 Stimulus material

The stimuli consisted of 305 3D objects from three existing databases (Downs et al., 2022; Peeters, 2018; Tromp et al., 2020). Since none of these databases alone provided enough semantically distinct objects for the study, they were combined. Five objects were used for training trials (see 2.3). The remaining 300 objects were divided into two size categories: half were small enough to fit in a shoebox at life size, while the other half were too large to fit into a shoebox (see behavioral task & Figure 1). The same stimulus set was used in two preceding studies. Participants enrolled in those studies were thus excluded from participation in the current study.

**Figure 1:**
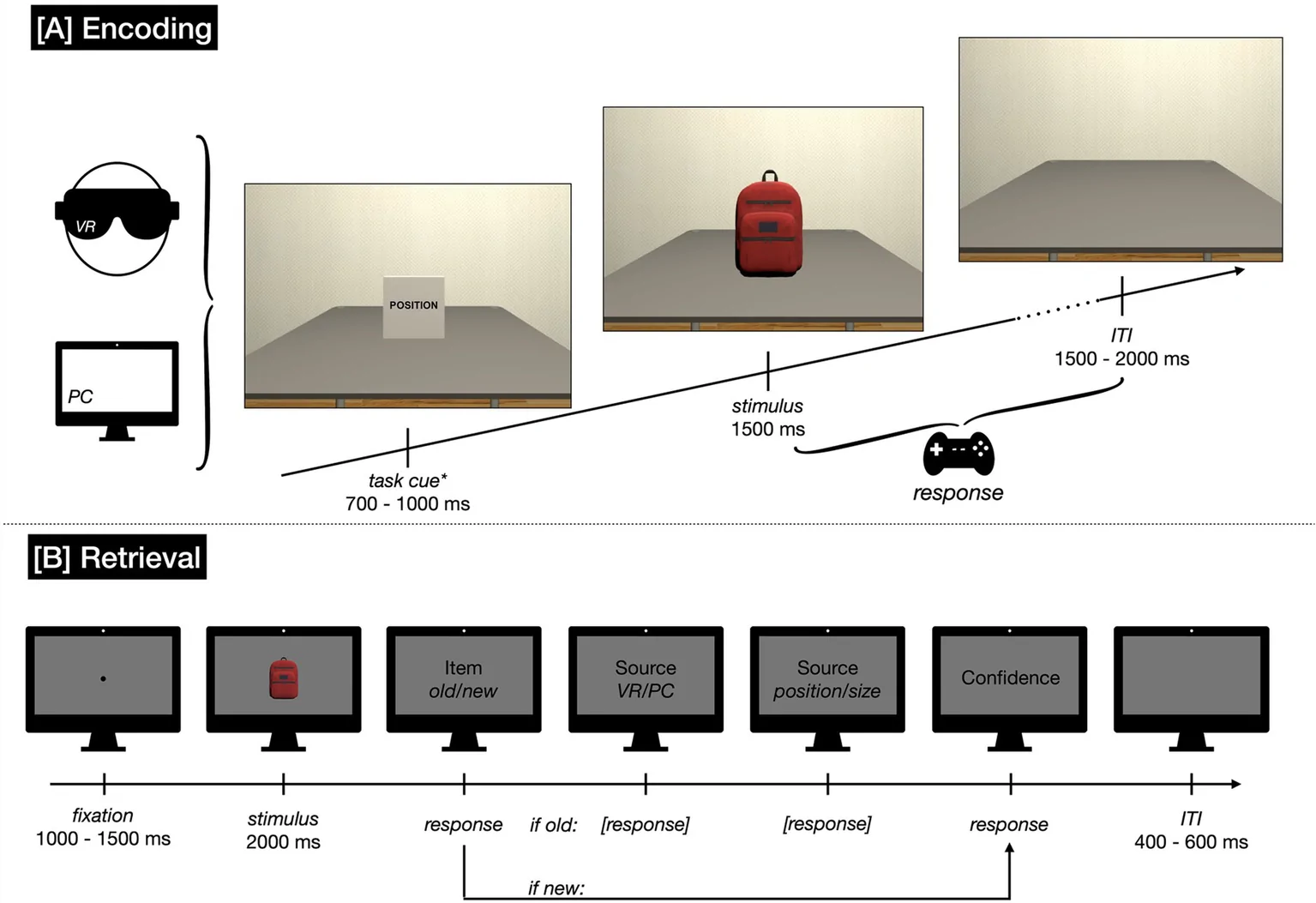
Schematic representation of the experimental procedure. **(A)** Participants encoded 100 stimuli each in VR and on a PC in a within-subjects design. The encoding phase took approximately 25 min, including two short breaks. *Task cue: The cue varied between “position” and “size”, instructing participants either to indicate the object’s position on the table or to estimate its real-life size. **(B)** The retrieval task took approximately 50 min, including two breaks. If participants rated an item as old, two source identification tasks were administered to assess the retrieval of the encoding modality (VR or PC) and the encoding task (size or position). ITI, Inter-trial-interval. This figure adapted from Kisker et al., 2025.

### 2.3 Procedure

The study follows up on a prior study from our working unit and addresses questions raised in that study (Kisker et al., 2025c). The methodology is therefore strongly based on the preceding study. Both studies adhered to established source memory designs, starting with an incidental encoding phase followed by an unannounced recognition memory test in a within-subjects design. The current study differs from the preceding study by linking source memory not only to the encoding modality but also to the encoding task.

The encoding phase comprised two conditions: a VR condition and a conventional desktop (PC) condition. Both conditions were presented in a counterbalanced order and were developed using Unity 5 (version 2023.2.1f1; Unity Technologies, San Francisco, USA). The virtual camera’s field of view was set to the default value of 60° in both conditions.

The VR condition was presented using an HTC Vive Pro 2 head-mounted display, providing a per-eye resolution of 2,448 × 2,448 pixels and a refresh rate of 90 Hz. Participants were positioned seated at a grey virtual table, 90 cm from the stimulus presentation location. The PC condition was presented using a 55″ display positioned 70 cm from the participant, offering a resolution of 1920 × 1,080 pixels and a refresh rate of 60 Hz. The same virtual environment, including the table, was rendered in 2D for this condition. The visual angle was aligned across conditions using the size of the fixation cube as a reference (see Figure 1). The cube was displayed on the desktop in the PC condition, measuring 15 cm x 15 cm, and in the VR condition, measuring 19 cm x 19 cm. For each condition, the distance between the participants and the cube was adjusted to achieve a viewing angle of 12°.

Each condition included five training trials and 100 experimental trials. For each participant, 300 stimuli from the databases were randomly divided into three subsets: 100 VR encoding trials, 100 PC encoding trials, and 100 distractor items for the recognition memory task. Within each subset, 50 objects were larger, and 50 were smaller than a standard shoebox, based on real-life size estimates. To keep the viewing angle constant, objects presented closer to the participant than the fixation cube (front position) were scaled relatively smaller, and objects presented farther away than the fixation cube (rear position) were scaled relatively larger relative to the fixation cube. Positions were randomized so that 50% of the objects larger or smaller than a shoebox were presented in the front position and 50% in the back position. The order of the category combinations was fully randomized.

Before the experimental trials, participants were verbally instructed, and any task-related uncertainties were addressed. To mitigate potential fatigue and muscular strain, a two-minute break was automatically scheduled after every 50 experimental trials. Each trial started with a task cue (700–1,000 ms), followed by stimulus presentation (1,500 ms) and an inter-trial interval (ITI, 1500–2000 ms; see Figure 1). In each trial, participants were asked to complete one of two possible encoding tasks. In 50% of the trials, the participants were cued to indicate as quickly as possible whether the presented object’s real-world size was larger or smaller than a conventional shoebox. Vice versa, participants were cued to indicate as quickly as possible whether the object’s position was the front one or the rear one in 50% of the trials. The order of the encoding task was randomized across all trials. Responses were given by pressing the shoulder buttons on a standard gamepad. Trials were paused until a response was registered, even if the stimulus had previously disappeared. The training initiation, the start of the experimental trial, and the break offset were signaled visually by a green cube displaying a start symbol. Training completion, break onset, and the experiment’s end were indicated by a red cube depicting a pause symbol. Each condition required approximately 12 min to complete, including the scheduled break.

After participants completed both encoding conditions, they were relocated to the electroencephalogram (EEG) laboratory. To minimize the potential confounding effects of rehearsal on subsequent recognition, participants completed a digit span task. For five minutes, participants were presented with sequences of single-digit numbers, starting at two digits. They were asked to memorize and freely recall each sequence by entering it on a keypad. Once three spans in a row were reproduced correctly, the length was increased by one digit. Once two sequences in a row were answered incorrectly, the length was reduced by one. The length could not fall below two digits. On average, participants completed 24.35 sequences, of which 75% were correct. The average length across completed sequences was 4.90, while the longest sequence reached was, on average, 6.81.

A 128-channel EEG was recorded during a recognition memory task, with participants seated inside a Faraday cage. Instructions were provided in writing on the screen, and participants had the opportunity to clarify any questions before the experimental trials began. The unannounced recognition test involved 2D images of all 300 3D objects. Objects encoded in VR are labeled “VR,” those encoded on the PC screen as “PC,” and new objects only shown during the retrieval phase are labeled “CR” (correctly rejected items). All stimuli were presented on a standard 24-inch monitor (1,020 × 1,200 pixels) with a grey background matching the color of the encoding table. The participants viewed the stimuli at horizontal and vertical angles of 7.96° and 3.49 °, respectively.

Each trial of the recognition task began with a 1,000–1,500 ms fixation period, followed by 2000 ms of stimulus presentation. Upon stimulus offset, a series of rating scales was presented sequentially (see Figure 1, Retrieval). Participants initially indicated stimulus recognition via an old/new judgment (item memory). For stimuli identified as “old,” two subsequent source-identification tasks were administered. Participants first indicated whether they had encoded the item in the VR or PC condition (source memory; source modality task) and then indicated whether they had performed the size or position task during encoding (source memory; source encoding task). This order was kept constant to match the primary source judgment used in the preceding study. Stimuli categorized as “new” bypassed the source identification task. Finally, participants rated their confidence in their response on a 4-point Likert scale (1 = very unsure, 2 = unsure, 3 = sure, 4 = very sure). The confidence rating referred to the participant’s subjective certainty regarding the full response sequence of the respective trial. Importantly, the source ratings were presented only after the recognition cue had disappeared and after the old/new judgment. Thus, participants were not instructed before or during object presentation to retrieve either the encoding modality or the encoding task. The series of ratings was followed by a 400–600 ms ITI (blank screen). The entire recognition task required approximately 50 min. To mitigate fatigue, participants were offered a break after every 100 trials. Breaks following 100 and 200 trials averaged 31 s and 44 s, respectively (average total break duration: 75 s). Upon completion of all trials, participants were asked to rate the subjective difficulty of the recognition task (0 = extremely easy, 10 = extremely difficult). The VR and EEG equipment were removed, and participants were debriefed.

### 2.4 Behavioral data

#### 2.4.1 Encoding phase

Response time and accuracy during encoding were assessed separately for the VR and PC conditions and for the encoding tasks (position, size). Response time was defined as the duration from stimulus onset to the participant’s response in milliseconds. Accuracy was calculated as the ratio of correct responses to the total responses in the encoding task.

#### 2.4.2 Retrieval phase

To determine the effect of encoding modality on memory retrieval, we quantified item memory performance by calculating the proportion of correctly identified old items for both VR- and PC-encoded stimuli. Criterion *C* was calculated based on these proportions [C = −0.5 * (z(hits) + z(false positive responses)), Hautus et al., 2021; Macmillan, 2002] to control for potential response biases. This measure indicates whether participants tend to give a particular answer if uncertain. Negative values of *C* indicate a tendency toward higher hit rates and false alarm rates (liberal biases). Positive values indicate a conservative response bias, characterized by a reduced tendency to respond ‘old’ and therefore typically associated with fewer false alarms but more misses (Macmillan, 2002).

D-prime (*d’*) was calculated as a measure of sensitivity, that is, the participant’s ability to distinguish between previously seen items and novel distractors (*d’* = *z(hits) – z(false alarms);* Swets et al., 1961). Source memory performance was determined as the ratio of correct source attributions to total source ratings for each modality and source identification task. In more detail, source-memory performance was calculated separately for VR- and PC-encoded items as the number of correctly identified source responses divided by the total number of source responses. Misses did not receive a source judgment and were therefore not included in this denominator. False alarms were rated as false source judgments.

Congruent with the preceding studies, further analyses examined confidence ratings associated with judgments made during the source identification task by means of encoding modality, contrasting conditions representing correct source attribution of PC-traces (PC-CS), incorrect source attribution of PC-traces (PC-FS), correct source attribution of VR-traces (VR-CS), incorrect source attribution of VR-traces (VR-FS), and correct rejections (CR) of new stimuli.

### 2.5 Electrophysiological recordings and preprocessing

An EEG was recorded using 128 active Biosemi electrodes (Amsterdam, Netherlands) during the recognition task. Four additional electrodes were used to measure the vertical and horizontal electrooculogram. Electrode placement followed the international 10–20 system. Additional CMS and DRL electrodes served as an online reference and ground. To ensure a good signal-to-noise ratio, the live signal was checked for line noise and slow drifts and corrected prior to the retrieval task. Data was sampled at 512 Hz, with an online bandpass filter of 0.016–100 Hz applied during recording. The EEG data were preprocessed in MATLAB (R2024a, MathWorks) and EEGLAB (version 2024, Delorme and Makeig, 2004).

The continuous EEG data were band-pass filtered from 0.25 to 30 Hz using EEGLAB’s pop_eegfiltnew function. Filter order and transition bandwidth were determined automatically using the function’s default settings. The high-pass filter was selected based on the literature recommending cutoff frequencies below 0.3 Hz, as higher cutoffs can affect slower ERP components such as the LPC and LPN, e.g., through artifactual amplitude loss (Tanner et al., 2015; Zhang et al., 2024). Bad channels were identified using Artifact Subspace Reconstruction (ASR; default settings; Mullen et al., 2015). The identified channels were interpolated (1.55 ± 2.35 channels per participant on average). Each electrode was detrended separately using the standard function *detrend* (MathWorks, Inc.) before the data were segmented into epochs from −500 ms to 2000 ms time-locked to the onset of the retrieval cue. A baseline correction from −500 ms to stimulus onset was applied. No flatline channels were detected. Epochs containing blinks were detected and removed using statistical control of artifacts in dense array studies (*SCADS*; Junghöfer et al., 2000), and the remaining trials were visually inspected for remaining blinks. On average, 105.71 ± 36.36 trials were excluded per participant due to blinks. An independent component analysis (ICA) was performed, and the ICLabel function (Pion-Tonachini et al., 2019) was used to identify and remove artifacts labeled as eye (> 0.85), muscle (> 0.9), or ECG (> 0.9). After excluding the respective components (*M* = 3.29 ± 5.73 *SD*) and the EOG channels, the data were re-referenced to the average reference. For the item memory task, trials were split into separate files containing trials in which PC objects were correctly identified as old (PC, 36.5 ± 12.2 trials), VR objects were correctly identified as old (VR, 36.7 ± 10.7 trials), and new objects were correctly rejected (CR, 54.3 ± 11.0 trials), respectively. Depending on participants’ responses, the number of trials per condition varied (please see below for information on signal-to-noise ratio and Supplementary material 1).

Time windows and regions of interest were derived from a current review and the preceding study, indicating an old/new effect in the time window from 300–500 ms after stimulus onset at frontal sensors (FN400) and a parietal old/new effect from 500–800 ms after stimulus onset at parietal sensors (LPC; Kamp et al., 2025; Kwon et al., 2023; Mecklinger, 2000; Mecklinger et al., 2016; Rugg and Curran, 2007). Based on the root-mean-square (RMS), the time window for the FN400 was shifted by 50 ms to 350–550 ms.

Typically, the FN400 is maximal around Fz, and the LPC is maximal around Pz or POz. The regions of interest were thus chosen based on the aforementioned literature, and thus, on the hypotheses. Because several electrodes located between conventional 10–20 positions in the 128-channel montage lack corresponding 10–20 labels, these additional electrodes cannot be specified using standard 10–20 nomenclature. Their spatial locations and inclusion in the respective clusters are therefore shown explicitly in Figures 2, 3. For the analyses of the FN400, electrodes Fz, F1, F2, FC1, FC2, AFz, and seven neighboring electrodes were averaged based on the regional maximum within the region of interest. Similarly, the electrodes Pz, P1, P3, P5, CP1, CP5, PO3, and seven neighboring electrodes were averaged for LPC analysis.

**Figure 2:**
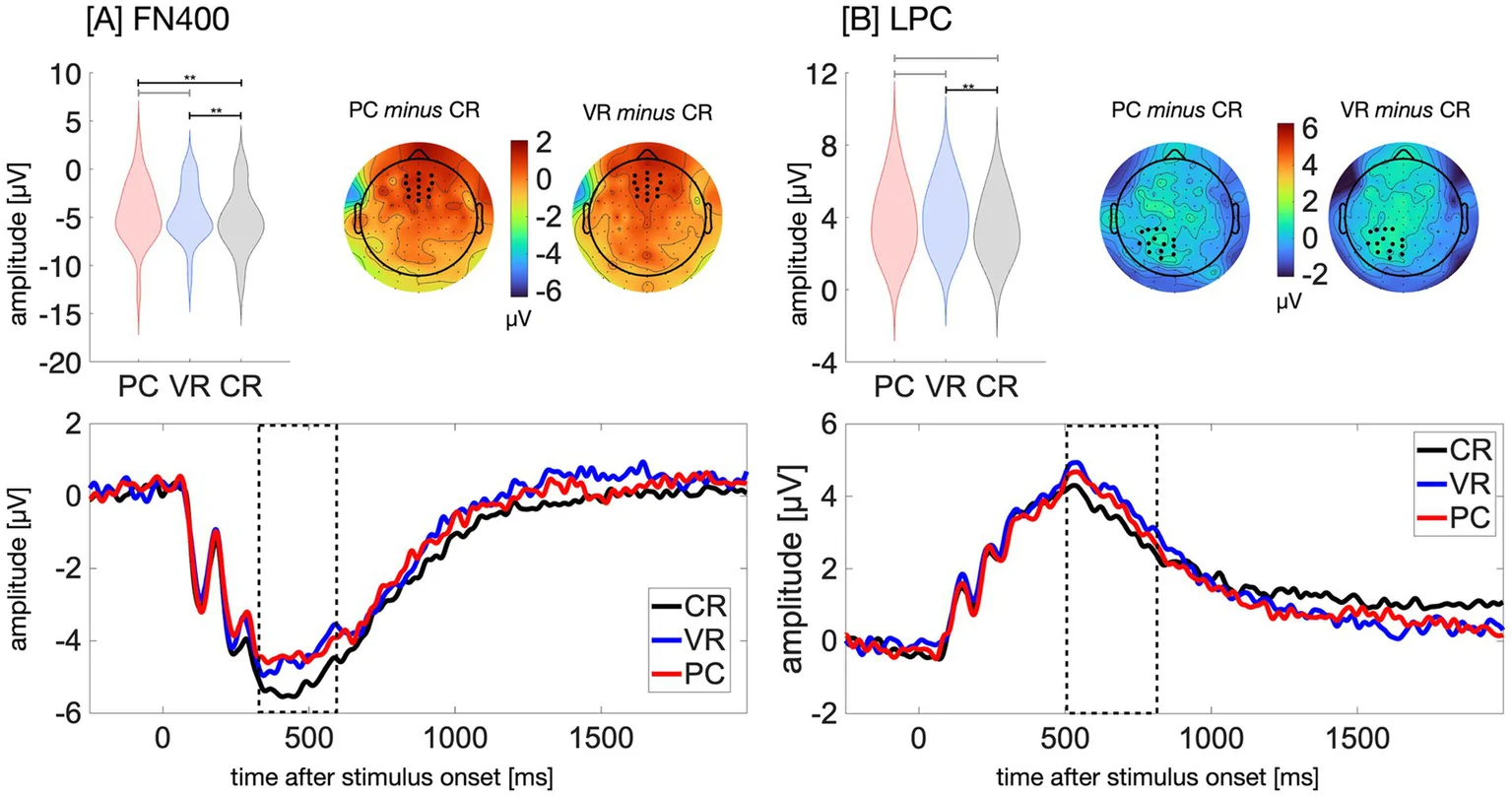
Topographical amplitude distribution and line plots of the FN400 and LPC responses for the old/new task. The black dots in the topographies indicate the electrodes averaged for statistical comparisons across conditions, and the grey dotted lines indicate the analyzed time windows. The violin plots depict the distribution of participant-level mean ERP amplitudes for each condition within the respective electrode cluster and analysis window (FN400: *n* = 28; LPC: *n* = 26). ** *p* ≤ 0.006, *** *p* < 0.001.

**Figure 3:**
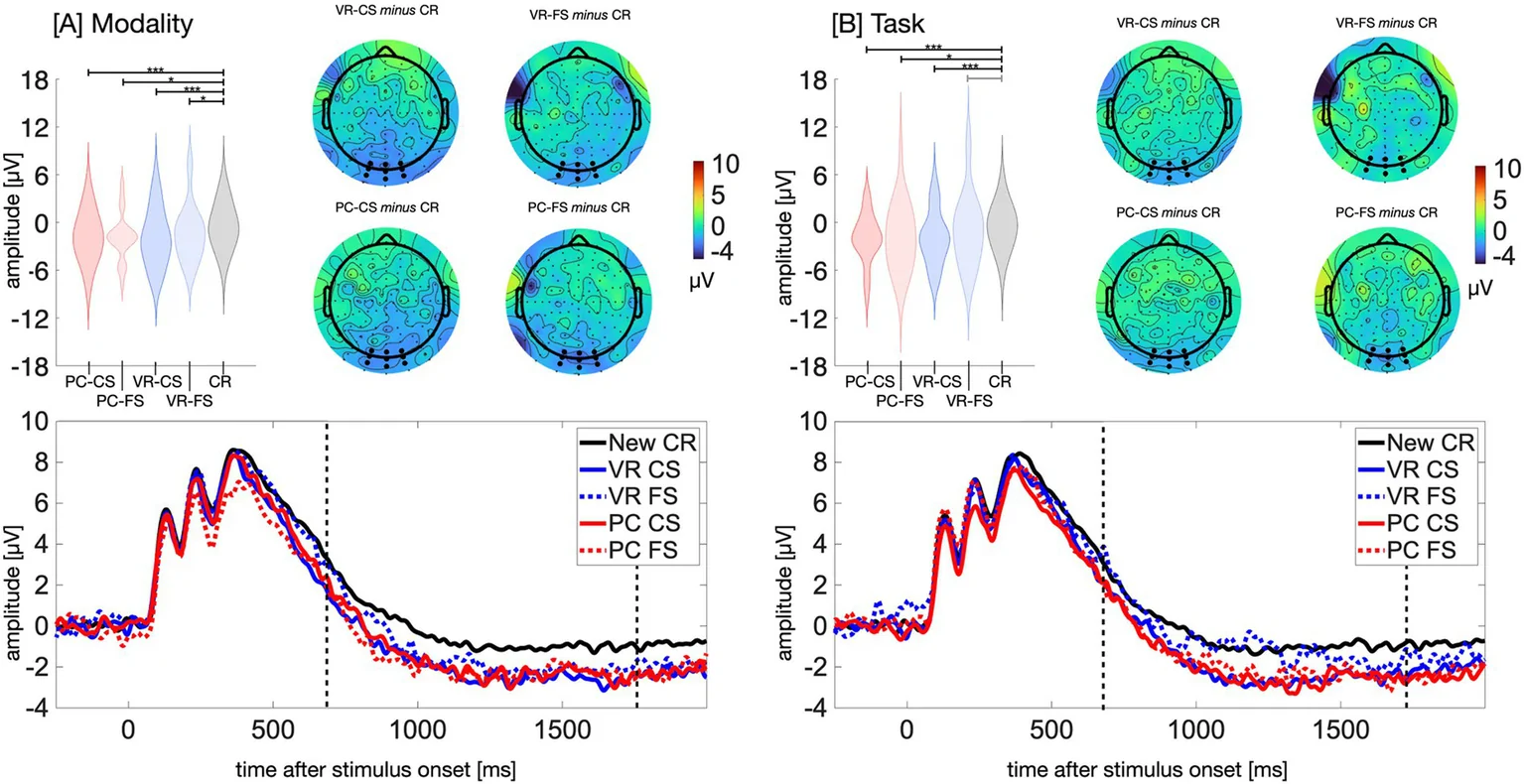
Topographical amplitude distribution and line plots of the FN400 and LPC responses for the old/new task. Panel **[A]** depicts the FN400 response, while panel **[B]** depicts the LPC response. The black dots in the topographies indicate the electrodes averaged for statistical comparisons across conditions, and the grey dotted lines indicate the analyzed time windows. The violin plots depict the distribution of participant-level mean ERP amplitudes for each condition within the respective electrode cluster and analysis window (FN400: *n* = 28; LPC: *n* = 26). **p ≤ 0.006, ***p < 0.001.

For the source identification task, trials were split into files for VR objects with correct (VR-CS) or false source attribution (VR-FS), PC objects with correct (PC-CS) or false source attribution (PC-FS), and correct rejections of new objects (CR; see Supplementary material for trial numbers). For source identification in the encoding tasks, false and correct identifications were collapsed across both tasks because separate analyses did not yield sufficient trials. The time window and region of interest were derived from previous literature, and thus our hypotheses, suggesting a source memory effect in the LPN from 700–1,200 ms and 1,200–1800 ms after stimulus onset and maximal around Pz and POz (Johansson and Mecklinger, 2003; Mecklinger et al., 2007, 2016), but has also been observed lateralized around P4 (Kamp et al., 2025; Kisker et al., 2025c). Although these electrodes guided the initial hypothesis, the final electrode cluster did not correspond to the *a priori* specified electrodes. Inspection of the scalp distribution in the present dataset revealed a local negative maximum over occipital sites across conditions. Based on this data-driven observation within the broader posterior region of interest, the final cluster comprised Oz, O1, O2, and six neighboring electrodes, including the electrode row between POz and Oz. Thus, the cluster was centered occipitally but extended into the parieto-occipital region. This distribution is consistent with previous descriptions of the LPN as a posterior or parieto-occipital negativity, including reports of occipital maxima or analyses incorporating occipital electrodes (Johansson and Mecklinger, 2003; Sommer et al., 2018). Accordingly, the observed local minimum was considered consistent with the LPN because it occurred in the expected late retrieval interval, was elicited in the context of source-memory processing, and showed a posterior/parieto-occipital scalp distribution. The precise scalp distribution of the LPN should be interpreted as exploratory, whereas the time windows, region of interest, and planned contrasts were hypothesis-guided.

To ensure a good signal-to-noise ratio, we calculated the Standardized Measurement Error (SME) for the mean amplitude of each selected electrode cluster within the time window of interest (Luck et al., 2021). The SME provides a measure of the precision of an individual’s ERP amplitude by estimating the standard error of the mean based on single trials before averaging. Rather than excluding participants based on a rigid trial-count threshold, we excluded individuals with high measurement noise, defined as an SME exceeding the group mean by more than two standard deviations in at least one condition. This approach ensures that the observed effects are not driven by trial-to-trial variability, even in conditions with limited numbers of trials. This SME-based procedure was thus used to account for the relatively few artifact-free trials in some source-sorted conditions and to base participant inclusion on the precision of the resulting ERP estimates rather than on trial counts alone. To countercheck that participants with low trial counts did not bias the group variance estimate, homogeneity of variance was compared across conditions at different trial limits, confirming that including all remaining subjects did not increase variance between conditions. Consequently, the analyses of the FN400 are based on 28 datasets, the LPC analyses on 26 datasets, and the LPN analyses on 25 datasets. Please refer to the Supplementary material for a detailed report of participant overlaps (Supplementary Table S1), as well as trial numbers and SME values per analysis (Supplementary Table S2).

### 2.6 Statistical analyses

According to the hypotheses, confirmatory analyses tested whether behavioral memory performance, as well as FN400 and LPC old/new effects differed between VR- and PC-encoded items. The LPN analyses were hypothesis-guided analyses in which the same retrieval-cue-locked ERP responses were sorted according to later modality-source and task-source accuracy. In contrast, analyses of confidence ratings, response times, and encoding accuracy were treated as secondary analyses.

As the general approach, inferential statistics were conducted, and effect sizes, partial eta squared (*η_p_^2^*), and Cohen’s *d* were reported. Additionally, *post hoc t*-tests were followed up by calculating the Bayes Factor (*BF_10_*) for the respective comparisons using JASP (JASP Team, 2024; Kelter, 2020) to provide more robust conclusions regarding differences or similarities between conditions. The default zero-centered Cauchy prior on the standardized effect size (scale r = 0.707) was used. The applied thresholds follow JASP’s recommendations (Kelter, 2020): *BF_10_* < 1 indicates anecdotal evidence for the H0; *BF_10_* < 0.33 signifies moderate evidence; *BF_10_* < 0.01 signals strong evidence; *BF_10_* < 0.03 shows very strong evidence for the H0. Conversely, *BF_10_* > 1 suggests anecdotal evidence for the H1; *BF_10_* > 3 indicates moderate evidence; *BF_10_* > 10 corresponds to strong evidence; and *BF_10_* > 100 reflects very strong evidence for the H1.

#### 2.6.1 Behavioral data

Response time and accuracy during the encoding phase were calculated separately for the VR and PC encoding conditions. Response time was measured as the time between stimulus onset and the participant’s response in milliseconds. Response times during encoding were compared using a 2 × 2 × 2 rmANOVA with the factors *Encoding Modality* (VR, PC), *Encoding Task* (size, position), and *Response* (correct response, false response). Eleven participants were automatically excluded because they provided no incorrect answers in at least one condition. Crucially, the effects of the factor *Encoding Modality* (i.e., its main effect and interactions) are particularly relevant to the research question. Effects that exist independently of the factor *Encoding Modality* would be reflected equally in both encoding conditions and would therefore not contribute to the differentiation of encoding modalities. For this reason, effects that do not involve the factor *Encoding Modality* were not followed up with *post hoc t*-tests. Post-hoc tests for the factor *Encoding Modality* were conducted as two-tailed paired-sample *t*-tests. Accuracy was quantified as the proportion of correct responses relative to all responses in the encoding task. It was analyzed using a 2 × 2 rmANOVA with the factors *Encoding Modality* (VR, PC) and *Encoding Task* (size, position). Post-hoc tests were conducted as two-tailed paired-sample *t*-tests.

The parameters operationalizing memory performance (see 2.4.2) were compared between VR and PC using one-tailed paired-sample *t*-tests. The significance level was Bonferroni-corrected to 0.013. Criterion *C* was compared by a two-tailed paired-sample *t*-test. With respect to the source identification testing for the retrieval of the encoding task, a 2 × 2 rmANOVA including the factors *Encoding Modality* (VR, PC) and *Encoding Task* (size, position) was carried out to account for potential interactions of both. Post-hoc tests were conducted as two-tailed paired-sample *t*-tests.

The participants’ confidence in their ratings was compared using a 1 × 5 rmANOVA with the within-factor *Condition* (VR-CS, VR-FS, PC-CS, PC-FS, CR), followed by two-tailed paired-sample *t*-tests if the rmANOVA indicated a significant effect of *Condition*. The significance level was Bonferroni-corrected to 0.006.

#### 2.6.2 Event-related potential

All ERP analyses were time-locked to the onset of the retrieval cue during the recognition phase. To analyze the old/new effects in the FN400 and the parietal LPC, a 1 × 3 rmANOVA with the within-subjects factor *Condition* (VR, PC, CR) was used for each ERP effect. If the rmANOVA indicated a significant effect of *Condition*, *post hoc t*-tests for paired samples were applied for comparisons between conditions. According to the hypotheses, the tests comparing both memory traces with correct rejections were one-tailed, whereas the comparison between both kinds of memory traces was two-tailed. The significance threshold for the *post hoc t*-test was corrected to ≤ 0.006 regarding the FN400 and to ≤ 0.005 regarding the LPC using the false discovery rate (FDR) according to the Benjamini-Hochberg method (Benjamini and Hochberg, 1995).

To analyze the LPN, separate 2 × 5 rmANOVAs with the within-factors *Time Window* (700–1,200 ms, 1,200–1800 ms) and *Condition* (VR-CS, VR-FS, PC-CS, PC-FS, CR) were conducted for the two source-classification schemes. In one analysis, trials were sorted according to later modality-source accuracy; in the other, the same retrieval-cue-locked ERP responses were sorted according to later task-source accuracy. Whenever necessary, the Greenhouse–Geisser correction was applied. In cases of a significant interaction between the two factors, a 1 × 5 RM ANOVA with only the factor *Condition* was conducted for each time window. In case of a significant main effect of *Condition* without a significant interaction, the amplitudes were averaged across the *Time Window* factor. In that case, mean amplitudes obtained separately for the 700–1,200-ms and 1,200–1800-ms intervals were averaged with equal weighting for subsequent comparisons.

The response to each old condition (VR-CS, VR-FS, PC-FS, PC-CS) was compared individually with the response to CR (see Gruber et al., 2008). Additionally, estimates of recollection and familiarity were calculated based on the most recent review (Kwon et al., 2023). In detail, stimuli correctly recognized as old and recalled with correct source attribution are considered to be based more strongly on recollection, which includes but goes beyond familiarity. In contrast, responses to stimuli correctly recognized as old but with incorrect source attribution are considered to predominantly reflect familiarity. Subtracting responses to stimuli with incorrect source attributions (FS) from those with correct source attributions (CS) was calculated as an index expected to be sensitive to recollection-related processing (VR-recollection, PC-recollection). Similarly, an index expected to be relatively sensitive to familiarity-related processing (VR-familiarity, PC-familiarity) was obtained by subtracting responses to correctly rejected stimuli (CR) from responses to stimuli with incorrect source attributions (FS). Importantly, these contrasts are not assumed to provide process-pure measures. Accordingly, the contrasts are interpreted as relative indices of recollection- and familiarity-related processing rather than as exclusive measures of either process. Based on the hypotheses, one-tailed *post hoc* paired-samples *t*-tests were conducted. Their significance threshold was FDR-corrected to ≤ 0.015 for both source identification tasks (encoding modality and encoding task). Effect sizes were calculated as partial eta squared (*η_p_^2^*) and Cohen’s *d*.

As an exploratory follow-up comparison, the VR-FS amplitudes averaged across time windows were compared between the two source types using a two-tailed paired-samples *t*-test. The *BF_10_* was calculated to quantify the relative evidence for the null and alternative hypotheses.

## 3 Results

### 3.1 Encoding phase

#### 3.1.1 Accuracy

Participants’ accuracy in the encoding task did not differ depending on the *Encoding Modality* [*F*(1, 30) < 0.01, *p* = 0.99, *η_p_^2^* < 0.001] but on the *Encoding Task* [*F*(1, 30) = 71.94, *p* < 0.001, *η_p_^2^* = 0.706]. The interaction of both factors did not reach significance [*F*(1, 30) = 0.19, *p* = 0.664, *η_p_^2^* = 0.006]. Based on the main effect of *Encoding Task*, participants achieved higher accuracy for the position task (94.05% ± 12.29) than for the size estimation task [76.68% ± 7.82%; *t*(30) = − 8.48, *p* < 0.001, *d* = 1.52; *BF_10_* = 6.346×10^+6^].

#### 3.1.2 Response time

The main effect of *Encoding Modality* did not reach statistical significance [*F*(1, 19) = 0.18, *p* = 0.679, *η_p_^2^* = 0.009]. Furthermore, all interactions involving *Encoding Modality* were non-significant (*Encoding Modality × Encoding Task: F*(1, 19) = 0.10, *p* = 0.754, *η_p_^2^* = 0.005; *Encoding Modality × Response: F*(1, 19) = 1.17, *p* = 0.293, *η_p_^2^* = 0.058; *Encoding Modality × Encoding Task × Response: F*(1, 19) = 0.80, *p* = 0.383, **η_p_^2^* = *0.040). These results indicate that RTs were invariant across the VR and PC encoding conditions and that *Encoding Modality* did not modulate the effects of task type or response correctness. In contrast, significant main effects were observed for *Encoding Task* [*F*(1, 19) = 24.65, *p* < 0.001, *η_p_^2^* = 0.565] and *Response* [*F*(1, 19) = 47.96, *p* < 0.001, *η_p_^2^* = 0.716]. These main effects were qualified by a significant two-way interaction between *Encoding Task* and *Response* [*F*(1, 19) = 5.25, *p* = 0.034, *η_p_^2^* = 0.216]. However, as outlined in the methods section, no post-hoc comparisons were conducted based solely on the latter factors. Descriptive statistics for all conditions are summarized in Table 1.

**Table 1:** Descriptive statistics for the response time (RT) in milliseconds after stimulus onset, depending on the encoding modality, encoding task, and correctness of the response.

Modality	Task	Response	Mean RT (ms)	SD (ms)
VR	Size	Correct	1407.05	350.77
		False	1629.16	566.64
	Position	Correct	1073.11	418.67
		False	1524.39	675.11
PC	Size	Correct	1400.72	415.55
		False	1617.39	494.11
	Position	Correct	1108.31	477.56
		False	1419.82	594.57

### 3.2 Behavioral memory performance

Overall, memory performance did not differ across encoding modalities but was relatively low. Participants correctly recognized approximately 51% of previously encoded objects regardless of the encoding modality [*t*(30) = −0.12, *p* = 0.453, *d* = −0.02; *BF_10_* = 0.19; *M*_VR_ = 51.23% ± 13.98% *SD*, *M*_PC_ = 51.45% ± 13.33% *SD*], and 86% (± 9.06% *SD*) of the new items were correctly rejected. The relatively low memory performance was reflected in participants’ perceived overall difficulty of the recognition task (*M* = 7.03 ± 1.33 *SD*; 0 = extremely easy, 10 = extremely difficult). Participants showed a conservative response bias which did not differ across encoding conditions as reflected in criterion *C* [*t*(30) = 0.15, *p* = 0.881, *d* = 0.03; *BF_10_* = 0.20; *M*_VR_ = 0.57 ± 0.32 *SD*, *M*_PC_ = 0.57 ± 0.31 *SD*]. Congruently, neither *d’* [*t*(30) = −0.12, *p* = 0.453, *d* = −0.02; *BF_10_* = 0.20; *M*_VR_ = 0.79 ± 0.36 *SD*, *M*_PC_ = 0.80 ± 0.35 *SD*] nor the percentage of correct source attributions of encoding modality differed between VR- and PC-engrams [*t*(30) = 0.58, *p* = 0.285, *d* = 0.10; *BF_10_* = 0.22; *M*_VR_ = 55.76% ± 14.26% *SD*, *M*_PC_ = 53.87% ± 15.80% *SD*]. Regarding the accuracy of the identification of the encoding task as source, no significant effect of *Encoding Modality* [*F*(1,30) = 0.28, *p* = 0.603, *η_p_^2^* = 0.01], but an effect of *Encoding Task* [*F*(1,30) = 100.12, *p* < 0.001, *η_p_^2^* = 0.77] was observed. The Encoding *Modality* × *Encoding Task* interaction did not reach significance [*F*(1,30) = 1.34, *p* = 0.256, *η_p_^2^* = 0.04]. Based on the main effect of *Encoding Task*, *post hoc t*-tests revealed that participants more frequently correctly identified the size task as the source (79.90% ± 11.00% *SD*) compared with the position task (54.50% ± 12.50% *SD*; *t*(30) = 10.01, *p* < 0.001; *d* = 1.80; *BF_10_* = 2.136×10 + 8).

However, participants’ confidence in their ratings differed between conditions in the source identification task of *Encoding Modality* [*F*(2.95, 88.35) = 10.51, *p* < 0.001, *η_p_^2^* = 0.26]. Participants were most confident in correctly rejected items and VR-items recalled with the correct source, and, surprisingly, PC-items recalled with the false source. They were followed by PC-traces retrieved with the correct source, and they were least confident about VR-items recalled with the incorrect source. The comparisons between PC-traces with incorrect source attribution and both PC-traces with correct source attribution and VR-traces with incorrect source attribution did not reach significance. Please refer to Figure 4 and Table 2 for the full report of the statistics.

**Figure 4:**
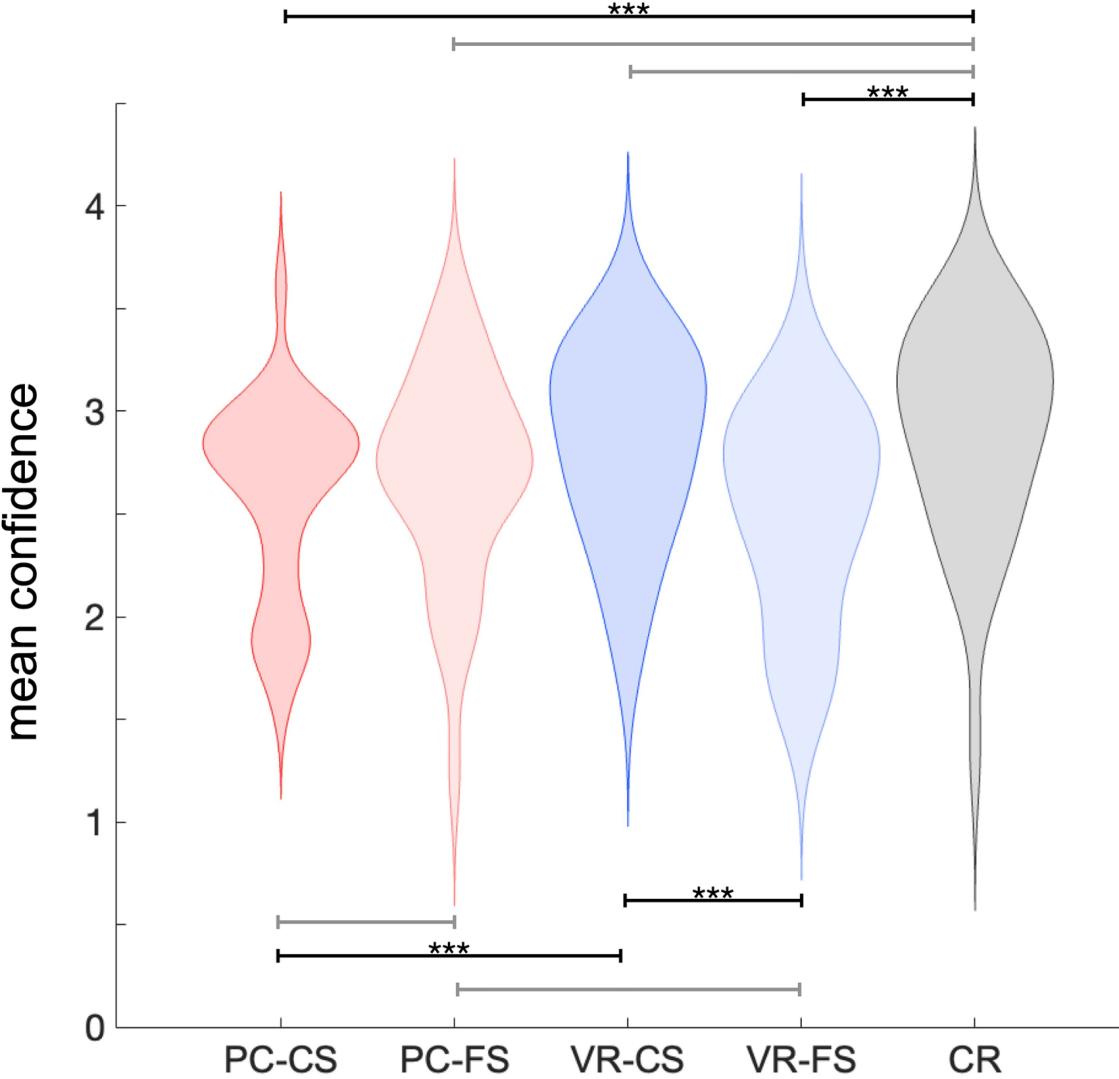
Participants’ mean confidence in their ratings across conditions of the source identification of the encoding modality. The confidence scale was a 4-point Likert scale ranging from very unsure [1] to very sure [4]. Comparisons significant after Bonferroni-correction (*p* < 0.006) are marked with ** *p* < 0.006, *** *p* < 0.001. Light-gray connecting lines indicate comparisons that did not reach significance after correction for multiple comparisons. The violin plots depict the distribution of participant-level mean for each condition (*n* = 31, see Table 2 for test statistics).

**Table 2:** Test statistics for participants’ confidence ratings based on the source identification of the encoding modality.

Two-tailed paired-samples *t*-tests				
Comparisons	*t*(30)	*p*	|d|	BF_10_
CR vs. VR-CS	−0.990	0.330	0.18	0.30
CR vs. VR-FS	−4.907	<0.001	0.88	762.82
CR vs. PC-CS	−3.914	<0.001	0.70	62.62
CR vs. PC-FS	−2.864	0.008	0.51	5.65
VR-CS vs. VR-FS	4.228	<0.001	0.76	135.99
PC-CS vs. PC-FS	−1.451	0.157	0.26	0.49
VR-CS vs. PC-CS	3.510	0.001	0.63	23.84
VR-FS vs. PC-FS	−2.329	0.027	0.78	1.96

The significance level was adjusted using the Bonferroni correction to 0.006. The reported tests were carried out using two-tailed tests.

### 3.3 Event-related potentials

#### 3.3.1 Old/New task

Significant effects of *Condition* were observed for both the early, frontally distributed old/new effect (FN400, *F*(2, 54) = 5.46, *p* = 0.007, *η_p_^2^* = 0.17) and the later, parietal old/new effect (LPC, *F*(2, 50) = 3.62, *p* = 0.034, *η_p_^2^* = 0.13; see Figure 2). The FN400 showed a classical old/new effect, revealing a less negative response to VR-traces [*t*(27) = 2.73, *p* = 0.006, *d* = 0.52; *BF_10_* = 4.22] and PC-traces [*t*(27) = 3.13, *p* = 0.002, *d* = 0.59; *BF_10_* = 9.82] compared to CR. A significant old/new effect in the LPC was also observed between VR-traces and CR [*t*(25) = 2.78, *p* = 0.005, *d* = 0.55; *BF_10_* = 4.67]. However, the old/new effect between PC-traces and CR did not reach significance [*t*(25) = 1.68, *p* = 0.053, *d* = 0.33], yet the BF indicated only anecdotal evidence for the H0 (*BF_10_* = 0.71). Neither the FN400 response nor the LPC response differed significantly between VR-traces and PC-traces [FN400: *t*(27) = 0.32, *p* = 0.752, *d* = 0.06; *BF_10_* = 0.21; LPC: *t*(25) = −0.91, *p* = 0.372, *d* = −0.18; *BF_10_* = 0.30]. The mean VR–PC amplitude difference in the FN400 response was −0.10 μV [95% CI: (−0.73, 0.53)], and 0.22 μV [95% CI: (−0.28, 0.71)] for the LPC response. In the course of the revision, a sensitivity analysis using the originally specified 300–500 ms FN400 window yielded the same pattern of results [*F*(2, 54) = 5.91, *p* = 0.010], with significant old/new effects for both PC [*t*(27) = 3.06, *p* = 0.005], and VR [*t*(27) = 2.40, *p* = 0.023], but no direct VR–PC difference [*t*(27) = −0.71, *p* = 0.484].

#### 3.3.2 Source identification of modality

The 2 × 5 rmANOVA yielded significant main effects of *Time Window* [*F*(1, 24) = 18.07, *p* < 0.001, *η_p_^2^* = 0.43] and *Condition* [*F*(3.04, 72.89) = 3.65, *p* = 0.008, *η_p_^2^* = 0.13; see Figure 3]. Since no significant interaction of both factors was found [*F*(3.02, 72.39) = 2.26, *p* = 0.088, *η_p_^2^* = 0.09], the amplitudes were averaged across the factor *Time Window* for post-hoc *t*-tests based on the main effect *Condition*. The LPN was significantly more negative across all kinds of memory traces compared with correct rejections (see Table 3). No significant differences were found regarding the estimates of recollection and familiarity (see Table 3).

**Table 3:** Test statistics for the LPN response for both source identification tasks.

One-tailed paired-samples *t*-tests					
Source type	Comparisons	*t*(30)	*p*	|d|	BF_10_
Modality	VR-CS vs. CR	−4.75	<0.001	0.95	335.34
	VR-FS vs. CR	−2.30	0.015	0.46	1.89
	PC-CS vs. CR	−3.81	<0.001	0.76	40.15
	PC-FS vs. CR	−3.00	0.003	0.62	8.56
	VR-Recollection vs. VR-Familiarity	0.48	0.319	0.10	0.23
	PC- Recollection vs. PC- Familiarity	1.814	0.041	0.36	0.87
	VR-Recollection vs. PC- Recollection	1.161	0.129	0.23	0.39
	VR-Familiarity vs. PC- Familiarity	−0.907	0.187	0.18	0.31
task	VR-CS vs. CR	−4.50	<0.001	0.90	190.42
	VR-FS vs. CR	−0.55	0.294	0.11	0.24
	PC-CS vs. CR	−3.98	<0.001	0.80	57.95
	PC-FS vs. CR	−2.31	0.015	0.46	1.94
	VR-Recollection vs. VR-Familiarity	−0.69	0.249	0.14	0.26
	PC- Recollection vs. PC- Familiarity	0.97	0.171	0.19	0.22
	VR-Recollection vs. PC- Recollection	−1.34	0.096	0.27	0.26
	VR-Familiarity vs. PC- Familiarity	1.51	0.073	0.30	0.47

The reported *p*-values are one-tailed. The significance threshold was FDR-corrected to ≤ 0.015 for both source identification tasks.

#### 3.3.3 Source identification of task

The 2 × 5 rmANOVA yielded significant main effects of *Time Window* [*F*(1, 24) = 17.94, *p* < 0.001, *η_p_^2^* = 0.43] and *Condition F*] (2.48, 59.63) = 2.99, *p* = 0.047, *η_p_^2^* = 0.11; see Figure 3). Since no significant interaction of both factors was found [*F*(2.96, 71.07) = 0.46, *p* = 0.765, *η_p_^2^* = 0.02], the amplitudes were averaged across the factor *Time Window* for post-hoc *t*-tests based on the main effect *Condition*. When trials were sorted according to later task-source accuracy, LPN amplitudes were significantly more negative than correct rejections for PC-CS, PC-FS, and VR-CS traces (see Table 3). However, the corresponding comparison for VR-FS traces items did not reach significance [*t*(24) = −0.55, *p* = 0.294, *d* = −0.11; *BF_10_* = 0.24]. No significant differences were found regarding the contrasts estimating recollection and familiarity (see Table 3).

#### 3.3.4 Exploratory comparison of source types

Despite a descriptive trend, the exploratory comparison of VR-FS responses between the two source types did not reach statistical significance [*t*(24) = −1.76, *p* = 0.092, *d* = −0.35, *BF_10_ =* 0.34]. The *BF_10_* provided anecdotal evidence for the null hypothesis. Thus, the data did not establish that VR-FS amplitudes differed between the two source types, while the evidence for their equivalence was also limited.

## 4 Discussion

The current study aimed to examine whether the encoding modality—Virtual Reality (VR) versus conventional monitor (PC)—is encoded as a defining contextual information of the memory trace, and can thus be subsequently retrieved bound to the stimulus. In particular, the current study addresses two limitations of a preceding study (Kisker et al., 2025c) by targeting processes that are not directly accessible through behavioral measures, such as non-criterial recollections, and by considering additional stages of neural activity associated with the retrieval of contextual information, namely the LPN. To this end, participants encoded stimuli in VR and on a PC, followed by an unannounced recognition memory test. The memory test adhered to established source memory designs: Participants were asked to indicate whether they remembered the objects from the encoding phase or considered them new (old/new task; item memory). In case of old decisions, it was additionally tested whether participants recalled having encoded the respective object in VR or on a PC (source identification of encoding modality; source memory) and whether they rated its real-life size or position during encoding (source identification of encoding task; source memory). As expected, participants’ memory performance did not differ as a function of encoding modality. Across encoding conditions, however, participants more frequently correctly remembered rating the object’s size during encoding than rating its position.

In contrast to the behavioral outcomes, the ERP results provided tentative indications of differences in retrieval-related processing. Both VR- and PC-encoded items elicited comparable FN400 old/new effects. In the LPC time window, an old/new effect was detected for VR-encoded items, whereas the corresponding comparison for PC-encoded items did not reach significance. Although the direct VR–PC comparison was not significant and therefore does not establish a reliable difference between modalities, the pattern aligns with previous findings suggesting that VR-based encoding may be associated with relatively greater recollection-related processing during retrieval. The source-sorted LPN analyses showed late effects for most old conditions. However, when trials were sorted by later task-source accuracy, the comparison between VR-traces attributed to the incorrect source and correct rejections did not reach statistical significance. Although this individual contrast differed descriptively between source types, the direct comparison did not establish a difference between both source types. The broadly convergent effects therefore constrain a modality-specific interpretation of the LPN, while smaller classification-dependent differences remain unresolved. Overall, our results indicate that VR-traces are not consistently associated with improved memory; yet they provide tentative evidence for differences in ERP correlates of retrieval. Despite limited direct VR–PC contrasts, the pattern is consistent with prior evidence that VR-based encoding may be associated with a different balance of familiarity- and recollection-related processes during recognition.

Our results align with the recurring pattern in comparisons of VR- and PC-based encoding, in which behavioral memory performance remains similar while the underlying memory processes differ in their magnitude (Kisker et al., 2021b, 2026; Kisker et al., 2025c). Specifically, participants’ performance in both the item and the source memory tasks was comparable across encoding conditions. Surprisingly, hit rates were relatively low, with participants correctly recognizing approximately 51% of old items, also reflected in relatively high perceived task difficulty. However, the high correct-rejection rate, positive *d′* values, and conservative response bias indicate that participants did not respond at chance. Together, the positive *d′* values and conservative response criterion indicate above-chance discrimination combined with a relatively cautious tendency to classify items as old. Descriptively, however, recognition rates were approximately 25% lower than in the preceding study (Kisker et al., 2025c). Given that the current study strongly builds on the preceding one, this difference is particularly striking. Potential explanations include (I) the encoding task, (II) the retention interval, or (III) subjective task difficulty.

- Unlike the previous study (Kisker et al., 2025c), the current encoding task alternated between rating the object’s real-life size and its position on the virtual table. Task switching is known to decrease subsequent memory performance, presumably due to impaired encoding of task-relevant information (Muhmenthaler and Meier, 2019) or divided attention (Craik et al., 1996). Since alternating the feature to be rated is a common standard in source memory paradigms (e.g., Gruber et al., 2008), we did not expect the encoding task to severely reduce memory performance, yet the encoding task switch may explain the differences in memory performance across studies.
- A further difference between studies concerns the retention interval. Due to the 128-channel EEG setup, the delay between encoding and retrieval was approximately 30 min longer than in the preceding study. However, previous pilot data comparing immediate retrieval with a 24-h delay found no differential effects of retention interval on VR- and PC-based encoding (Kisker et al., 2025c). Therefore, differences in delay are unlikely to explain the lower memory performance observed.
- Moreover, participants perceived the retrieval task as relatively difficult. Although good, their ability to distinguish between previously seen items and novel distractors (*d’*) was almost half of that in the preceding study. Since subjective difficulty was not assessed in the preceding study (Kisker et al., 2025c), no direct comparison is possible. At least, the indications of relatively high task difficulty in the current study suggest that task difficulty may have contributed to reduced memory performance. Accordingly, the findings should be interpreted as evidence about retrieval-related processing under relatively difficult retrieval conditions.

This relatively low hit rate also reduced the number of correctly recognized trials available for ERP analyses, particularly after sorting trials by source accuracy. However, the ERP analyses did not rely on trial numbers alone. We assessed the precision of the condition-specific ERP estimates using the standardized measurement error (SME) and excluded datasets with high measurement noise. This SME-based procedure helps maintain the stability of the retained ERP averages despite unequal and sometimes low trial numbers across conditions. Nevertheless, lower recognition performance may have reduced sensitivity to smaller ERP effects, especially for modality-specific differences.

From a behavioral perspective, previous studies reporting comparable memory performance despite differences in memory processes between VR- and PC-based encoding hypothesized that differences across encoding conditions might have been overshadowed by ceiling effects in relatively easy tasks (e.g., Johnsdorf et al., 2025; Kisker et al., 2021b). Our findings of similar memory performance in a relatively difficult task, despite low memory performance, challenge this hypothesis. At least for the retrieval of everyday objects, differences between the VR and PC conditions do not seem to depend solely on task difficulty. However, our study does not provide insight into whether such dependencies might be intertwined with factors relevant only to more complex stimuli or settings.

Beyond behavioral outcomes, neural activity patterns tentatively pointed toward differences in the roles of recognition subprocesses during retrieval across conditions. We replicated previous findings that the contribution of item familiarity, associated with the FN400 response, was consistent across conditions (Kisker et al., 2025c). In contrast, the later parietal old/new effect in the LPC was only significant for VR-traces relative to correct rejections, whereas the corresponding contrast for PC-traces did not reach significance. This pattern is generally consistent with previous findings associating VR-based encoding with recollection during retrieval. However, because the direct contrast between VR- and PC-traces was not significant, the present data do not independently provide evidence for a difference between the two encoding modalities. Rather, the findings suggest broadly comparable recognition-related processing, though aligning with previous indications that VR-based encoding may facilitate the retrieval of episodic information at later stages of processing.

Thus, although generally in line with previous associations of VR-based memory traces with recollection, the finding is somewhat surprising, as previous studies did not report modality-dependent effects on recollection-related responses but rather weaker familiarity-related responses (Johnsdorf et al., 2026; Kisker et al., 2026; Kisker et al., 2025c). Remarkably, the latter pattern was even evident in a recent study conducting a threefold comparison of retrieval after encoding on a PC, in VR, and in physical reality (Johnsdorf et al., 2026). While all encoding conditions exhibited similar LPC responses linked to recollection, only the PC condition exhibited a significant FN400 old/new effect linked to familiarity. Although the two studies show different patterns of results regarding the old/new effects, they align with the conclusion that familiarity-related processes play a smaller role than recollection in successful retrieval following VR-based encoding. Even more remarkably, the three-way comparison showed that VR-based encoding more precisely represents the magnitude of real-life processes, suggesting that it might closely mimic real-world processes (Johnsdorf et al., 2026; Kisker et al., 2025a; Kisker et al., 2025b).

Within the framework of dual-process accounts, the FN400 and LPC have been strongly associated with familiarity- and recollection-related retrieval processes, respectively. However, neither component provides a process-pure measure. The FN400 has also been discussed as reflecting conceptual fluency or an N400-like repetition effect rather than familiarity-based recognition alone (Voss et al., 2010, 2012; Voss and Paller, 2007). According to this approach, repeated presentation of stimuli during encoding and retrieval might result in conceptual fluency, which overlaps with familiarity in the FN400. At the same time, several aspects of the present design limit a strong fluency or repetition-based interpretation: each object was presented only once during encoding and retrieval, separated by the distraction task and EEG preparation. Moreover, if our findings were substantially affected by fluency, this should have had a stronger effect on the response to PC-encoded objects, given their greater perceptual overlap with the retrieval cues. However, the FN400 old/new effect did not differ between VR- and PC-encoded objects, which argues against a simple account based on perceptual overlap. Thus, fluency-related processing may have contributed to the FN400, but it is unlikely to fully explain the observed old/new pattern.

Similarly, although the LPC is commonly interpreted as recollection-sensitive, it can also vary with memory strength, confidence, and P3b-like decision-related activity (Brezis et al., 2017; Kwon et al., 2023; Sun et al., 2024). Hypothetically, lower memory performance or higher task difficulty might have enhanced the importance of recollection-based retrieval or memory strength. This could have facilitated divergent effects that earlier studies with overall higher performance did not detect. Alternatively, variations in memory confidence might have affected the current results, as the parietal old/new effect has been shown to covary with both memory strength and confidence (Brezis et al., 2017; Sun et al., 2024). Our results on the participants’ confidence provide an exploratory measure of subjective certainty for the combined retrieval response. However, the pattern of confidence ratings did not align with the LPC findings. While participants were most confident about correct rejections and for VR-traces with correct source attribution, even PC-traces with incorrect source attribution were associated with relatively high confidence despite the non-significance of the LPC effect. The present results suggest that confidence alone is unlikely to account for the LPC pattern, but the stimulus-locked design does not allow us to fully separate recollection-related activity from strength-, confidence-, or decision-related positivity. Our analytical approach, results, and interpretation most closely align with the recent meta-analysis by Kwon et al. (2023), which favors a strong but not exclusive sensitivity of the FN400 and LPC to familiarity- and recollection-related processing, respectively. However, further research is required to disentangle the mnemonic processes reflected by both ERP old/new effects.

Another explanation relates to the notion that VR experiences offer a more sensory-rich context than PC experiences (Parsons, 2015; Serino and Repetto, 2018; Smith, 2019; Smith and Mulligan, 2021). Our series of studies explicitly aimed to determine whether switching from 2D to 3D presentation – while maintaining the visual context, content, and fidelity per se – would result in different contributions of memory processes and different levels of retrieval performance. Consequently, the retrieval of VR-traces might include richer contextual details obtained during 3D encoding. Depending on the specific account, the search, the reconstruction, or the amount of contextual details available during retrieval has been linked to the LPN (Addante et al., 2024; Cycowicz et al., 2001; Herron, 2007; Johansson and Mecklinger, 2003; Kamp et al., 2025; Mecklinger et al., 2016). Accounting for previous limitations, our results provide insights into whether differences between VR- and PC-traces depend on the specific source information assessed and the specific time window used for LPN analyses. With respect to the early LPNs’ link to the immediate retrieval process and its link to subsequent source monitoring and post-retrieval processing in later stages, our data indicated a similar pattern of results across time windows rather than divergent effects.

More notably, we did not replicate previous findings indicating attenuated familiarity for VR-traces with incorrect source attribution (VR-FS) with respect to encoding modality. In the preceding study (Kisker et al., 2025c), several measures, including the LPN, did not differentiate between VR-traces with incorrect source attribution and correctly rejected new objects when tying source retrieval to encoding modality. These measures indicated attenuated contributions of familiarity and, vice versa, greater contributions of recollection to retrieval of VR-based memory traces, respectively, and were discussed with respect to context-transfer effects. Whereas PC-traces, as well as VR-traces recalled with correct source attribution, might include contextual details, such as the stimulus dimensionality, VR-traces with incorrect source attributions might not have bound this information during encoding, resulting in a greater mismatch with the retrieval cue. In contrast, the current results did not differentiate between VR-traces with incorrect source attribution and correctly rejected items when trials were sorted by later task-source accuracy, rather than by later modality-source accuracy. Although the pattern of results was not fully identical at the descriptive level, the comparison of the LPN response to VR-encoded items across source types did not reach statistical significance, whereas the Bayes Factor provided only anecdotal evidence of equivalence. Since the VR-FS condition has a similar number of trials on average as all further conditions with incorrect source attribution, as well as a comparable standardized measurement error (SME, Luck et al., 2021; see Supplementary material) across the conditions resulting from the encoding task identification, we consider it relatively unlikely that the results are due to insufficient measurement precision of this condition. However, the broadly convergent LPN effects suggest that the retrieval-cue-locked response was not uniquely associated with either subsequent source judgment, whereas smaller source-dependent differences remain unresolved. Assessing both source judgments therefore helped to constrain the interpretation of previous modality-based LPN effects (Kisker et al., 2025c), although the present results cannot determine whether their convergence reflects non-criterial recollection or more general retrieval-related processing.

One factor that may have contributed to the differences between the present LPN results and those reported in the preceding study is the task switch during encoding. Whereas the encoding modality was rather passively perceived in both studies and not immediately relevant to solving the encoding task, the latter might have afforded more cognitive resources due to the task switch, in contrast to the uniform encoding task in the preceding study. In turn, fewer cognitive resources might have been available to process information irrelevant to the acute task. In case fewer resources were available during encoding to process the stimulus modality, retrieving it might require greater effort. Since the LPN has been observed to be sensitive to retrieval effort, with greater effort yielding more negative responses (Johansson and Mecklinger, 2003; Mecklinger et al., 2007, 2016), even conditions with less contextual detail on dimensionality available might result in significantly more negative responses. Although the current LPN effects did not undermine this notion, the behavioral results indicate that task- and modality-related information differed in accessibility. The relatively high accuracy in correctly identifying the size task as a source particularly indicates that some information from encoding was more easily accessible than others. Congruently, Mollison and Curran (2012) distinguished between intrinsic and extrinsic source associations, where intrinsic features are inherent to the stimulus (e.g., stimulus color), whereas extrinsic features are bound to the surrounding context (e.g., background color). In the present paradigm, the stimulus’s dimensionality may be ambiguous with respect to this dichotomous notion. On the one hand, three-dimensional presentation provides perceptual cues, such as stereoscopic depth, that may facilitate evaluation of the encoding task and thus serve as an intrinsic feature. On the other hand, dimensionality may also be perceived as part of the overall context in which the stimulus is presented, particularly when cognitive resources during encoding are focused on the task itself. Under conditions of increased task demands, such as task switching, dimensionality may therefore be encoded less strongly stimulus-bound and instead function as an extrinsic contextual cue. However, disentangling this approach requires encoding three-dimensional stimuli within a two-dimensional context and vice versa. Consequently, our results thus raise the question of under which circumstances the retrieval of context dimensionality and intrinsic stimulus features may interact.

In addition to these encoding-related factors, the LPN effects may depend on retrieval conditions. A parallel follow-up study to the preceding study examined the dependence of the previous results on context-transfer effects (Kisker et al., 2026). In this study, retrieval of both VR- and PC-traces was conducted in a VR environment. While this study indicated that the observed results go beyond context-transfer effects, the LPN did not differ between conditions. Even more, the characteristic morphology of the LPN, which means a posterior negativity with an onset of 700–800 ms after stimulus onset, was not observed. In contrast, these morphological characteristics were evident in both the preceding and the current study, indicating that the sensitivity of the LPN might, to some degree, depend on the retrieval condition. To the best of our knowledge, the LPN has not yet been compared across different retrieval conditions under consistent encoding conditions, and thus remains an area to explore.

## 5 Conclusion

The present study examined whether the encoding modality (VR vs. PC) is encoded and retrieved as a defining characteristic of episodic context, and whether retrieval-related ERP activity differs when trials are sorted by different types of source information. Behavioral memory performance was comparable across encoding modalities and did not indicate a VR-related advantage in recognition or source attribution. At the neural level, FN400 old/new effects, often associated with familiarity, were similar for VR- and PC-encoded items. In the LPC time window, an old/new effect sensitive to recollection was detected for VR-encoded items, whereas the corresponding comparison for PC-encoded items did not reach significance. Although the direct VR–PC comparison was not significant, this pattern is generally consistent with previous findings linking VR-based encoding predominantly to recollection-related retrieval processes. At the same time, both source-sorted analyses yielded LPN effects for most conditions. Although the individual contrasts differed descriptively, the broadly convergent LPN effects across source types rather suggest that the retrieval-cue-locked response was not uniquely associated with either of the two subsequent source judgments. Thus, assessing both judgments helped to constrain a modality-specific interpretation of the LPN effects.

Overall, the findings tentatively support previous evidence that retrieval-related processes may differ between VR- and PC-encoded items. However, the present data do not establish a robust modality-specific shift in the balance of familiarity- and recollection-related processes. Rather, recognition-related processing appears largely comparable across encoding modalities, while the present ERP pattern remains compatible with the idea that VR-based encoding may support recollection-related access to contextual information under some retrieval conditions. Future studies should therefore directly test whether and under which retrieval conditions VR-based encoding is associated with stronger recollection-related activity than PC-based encoding, for example by manipulating retrieval orientation, task focus, and retrieval modality. Clarifying these conditions may also be relevant for applied settings, such as memory training in immersive environments, where the potential benefit of promoting recollection-related retrieval processes would need to be tested directly.

## 6 Limitations

Overall item memory performance was relatively low, with participants recognizing only about 51% of old objects. Although *d′* and criterion *C* indicated reliable but conservative discrimination between old and new items, low hit rates reduced the number of trials available for ERP analyses, particularly after sorting trials according to later source accuracy. To address this issue, ERP datasets were not selected based on trial counts alone but on the precision of condition-specific ERP estimates using the Standardized Measurement Error (SME; Luck et al., 2021). Participants whose SME exceeded two standard deviations above the group mean in at least one condition were excluded from the respective component-specific analysis. Moreover, although the *a priori* power estimate was likely optimistic for smaller modality-specific effects, the final ERP samples exceeded the estimated minimum by more than twofold and were consistent with common practice in within-subject ERP research (Clayson et al., 2019). Nevertheless, the study was primarily sensitive to relatively robust ERP effects, whereas smaller modality-specific differences may have gone undetected.

Another important design consideration is that participants were not instructed before or during the presentation of retrieval cues to retrieve either the encoding modality or the encoding task; both source judgments were made only after the old/new response. Therefore, the modality-source and task-source analyses represent two post-hoc classifications of the same retrieval-cue-locked ERP responses. The present findings should consequently be interpreted as ERP correlates of subsequent source-attribution accuracy rather than as evidence that different contextual retrieval demands directly modulated neural activity during the EEG interval. Future studies could address this issue more directly by cueing the relevant source dimension before stimulus onset or by using separate retrieval blocks that require modality-source versus task-source retrieval. In a similar vein, the two source judgments were presented in a fixed order to maintain consistency with the preceding study. The modality-source judgment always preceded the task-source judgment. Because the ERPs were time-locked to the retrieval cue, they preceded both source responses; however, the preceding modality-source judgment may have influenced the later task-source response at the behavioral level. Future studies might counterbalance the source-question order or cue source dimensions separately.

Moreover, stimulus dimensionality and context dimensionality were inseparable in the present design. Consequently, it remains unclear whether participants encoded the dimensionality of the stimulus itself, the surrounding environment, or both. To determine whether dimensionality is encoded as an intrinsic or extrinsic source of information, future research could explore encoding three-dimensional stimuli in a two-dimensional setting and vice versa.

## Data Availability

The datasets presented in this study can be found in online repositories. The datasets presented in this study are available in the online repository OSF: https://osf.io/vbxk7/overview?view_only=31851614627243f59a3403f9b89d820f. The repository contains an SPSS data file with participant-level aggregated values for all variables included in the reported analyses, as well as additional descriptive variables (e.g., demographic information and digit-span performance). The SPSS file includes detailed variable labels.
