# Supplementary material for: Electrophysiological correlates of contextual retrieval following virtual reality and desktop-based encoding
Source: Front Hum Neurosci. 2026 Oct 14;20:1866317. doi: 10.3389/fnhum.2026.1866317 (PMC13713475; doi:10.3389/fnhum.2026.1866317)
Supplement: Supplementary file 1 [file Table_1.DOCX]

Supplementary Material

**Supplementary Table S1**

*Participant exclusion across behavioral and ERP analyses.*

| *Participant No.* | *Behavioral Analyses* | *FN400 Analyses* | *LPC Analyses* | *LPN Analyses: Encoding Modality* | *LPN Analyses: Encoding Task* |
| --- | --- | --- | --- | --- | --- |
| 1 | 0 | 0 | 0 | 0 | 0 |
| 2 | 0 | 0 | 0 | 0 | 0 |
| 3 | 0 | 0 | 0 | 0 | 0 |
| 4 | 0 | 0 | 0 | 0 | 0 |
| 5 | 0 | 0 | 0 | 0 | 0 |
| 6 | 0 | 0 | 0 | 0 | 0 |
| 7 | 0 | 0 | 0 | 0 | 0 |
| 8 | 0 | 1 | 0 | 1 | 1 |
| 9 | 0 | 0 | 1 | 0 | 0 |
| 10 | 0 | 0 | 0 | 0 | 0 |
| 11 | 0 | 0 | 0 | 0 | 0 |
| 12 | 0 | 0 | 0 | 1 | 1 |
| 13 | 0 | 1 | 0 | 1 | 1 |
| 14 | 0 | 0 | 0 | 0 | 0 |
| 15 | 0 | 0 | 0 | 0 | 0 |
| 16 | 0 | 0 | 0 | 0 | 0 |
| 17 | 0 | 0 | 0 | 0 | 0 |
| 18 | 0 | 0 | 0 | 0 | 0 |
| 19 | 0 | 0 | 0 | 1 | 1 |
| 20 | 0 | 0 | 0 | 0 | 0 |
| 21 | 0 | 0 | 0 | 0 | 0 |
| 22 | 0 | 0 | 0 | 0 | 0 |
| 23 | 0 | 0 | 0 | 0 | 0 |
| 24 | 0 | 0 | 1 | 1 | 1 |
| 25 | 0 | 1 | 1 | 1 | 1 |
| 26 | 0 | 0 | 0 | 0 | 0 |
| 27 | 0 | 0 | 1 | 0 | 0 |
| 28 | 0 | 0 | 0 | 0 | 0 |
| 29 | 0 | 0 | 0 | 0 | 0 |
| 30 | 0 | 0 | 1 | 0 | 0 |
| 31 | 0 | 0 | 0 | 0 | 0 |

*Note*. 0 = participant included in the respective analysis; 1 = participant excluded. All 31 participants were included in the behavioral analyses. Following analysis-specific exclusion, the final samples comprised 28 participants for the FN400 analysis, 26 for the LPC analysis, and 25 for each of the two LPN analyses. The encoding-modality and encoding-task LPN analyses included the same 25 participants. Across ERP analyses, 22 participants were retained in all analyses. Pairwise overlap was 24 participants for the FN400 and LPC analyses, 25 for the FN400 and LPN analyses, and 22 for the LPC and LPN analyses.

**Supplementary Table S2**

*Condition-Specific Trial Counts and Standardized Measurement Error (SME) for the ERP Analyses*

|  |  |  | *Trials* | | *SME* |
| --- | --- | --- | --- | --- | --- |
| *Analysis* | *Condition* | *N* | *M ± SD* | *range* | *M ± SD* |
| FN400 | PC | 28 | 36.5 ± 12.2 | 13–68 | 0.91 ± 0.21 |
|  | VR | 28 | 36.7 ± 10.7 | 17–72 | 0.93 ± 0.20 |
|  | CR | 28 | 54.3 ± 11.0 | 39–88 | 0.76 ± 0.14 |
| LPC | PC | 26 | 35.7 ± 12.5 | 13–68 | 0.71 ± 0.17 |
|  | VR | 26 | 36.0 ± 12.1 | 10–72 | 0.73 ± 0.18 |
|  | CR | 26 | 54.4 ± 11.6 | 39–88 | 0.58 ± 0.14 |
| LPN, encoding modality | PC-CS | 25 | 23.1 ± 9.0 | 7–42 | 1.37 ± 0.48 |
|  | PC-FS | 25 | 16.0 ± 7.2 | 5–32 | 1.74 ± 0.57 |
|  | VR-CS | 25 | 20.3 ± 8.6 | 8–41 | 1.51 ± 0.53 |
|  | VR-FS | 25 | 18.6 ± 6.5 | 5–31 | 1.46 ± 0.45 |
|  | CR | 25 | 57.3 ± 13.1 | 37–88 | 0.86 ± 0.31 |
| LPN, encoding task | PC-CS | 25 | 25.3 ± 7.5 | 14–45 | 1.37 ± 0.48 |
|  | PC-FS | 25 | 11.4 ± 4.7 | 4–20 | 1.74 ± 0.57 |
|  | VR-CS | 25 | 26.2 ± 9.2 | 9–49 | 1.51 ± 0.53 |
|  | VR-FS | 25 | 11.4 ± 4.4 | 3–21 | 1.46 ± 0.45 |
|  | CR | 25 | 53.9 ± 10.7 | 33–84 | 0.86 ± 0.31 |

*Note.* Trial counts represent the number of artifact-free trials contributing to each ERP average. The slightly different CR trial counts between the two LPN classifications resulted from independent visual artifact inspection of separately retained datasets after automatic artifact correction; participant-level CR trial counts were highly correlated across datasets (*r* = .976, *p* < .001) and did not differ significantly (*t*(24) = 1.14, *p* = .264). The SME was calculated for the mean amplitude of the respective electrode cluster and analysis window based on single-trial variability. Participants with an SME exceeding the group mean by more than two standard deviations in at least one condition of the respective analysis were excluded before calculating the mean reported in this table. Final sample sizes were N = 28 for the FN400 analysis, N = 26 for the LPC analysis, and N = 25 for both LPN analyses. SME = standardized measurement error; PC = personal computer; VR = virtual reality; CR = correct rejection; CS = correct source attribution; FS = false source attribution.
